# Adiponectin deficiency contributes to the development and progression of benign prostatic hyperplasia in obesity

**DOI:** 10.1038/srep43771

**Published:** 2017-03-03

**Authors:** Shi Fu, Huan Xu, Meng Gu, Chong Liu, Qiong Wang, Xiang Wan, Yanbo Chen, Qi Chen, Yubing Peng, Zhikang Cai, Juan Zhou, Zhong Wang

**Affiliations:** 1 Department of Urology, Shanghai Ninth People’s Hospital, Shanghai Jiao Tong University School of Medicine (SJTUSM), Shanghai, 200011, China

## Abstract

The incidence of benign prostatic hyperplasia (BPH) is increasing among obese individuals, but few studies have fully explained the underlying mechanisms. We aimed to elucidate the relationship between obesity and BPH. Herein, we show that in prostatic epithelial and stromal cells, adiponectin exerts multifunctional effects including anti-proliferation, blocking of G1/S-phase progression and the promotion of apoptosis via inhibiting the MEK-ERK-p90RSK axis. Furthermore, we found that a high-fat diet (HFD) led to adiponectin deficiency and microscopic BPH in a mouse model of obesity. And an adiponectin supplement protected the obese mice from microscopic BPH. The present study provides evidence that adiponectin is a protective regulator in the development and progression of BPH and that adiponectin deficiency causally links BPH with obesity.

Benign prostatic hyperplasia (BPH) is a prevalent pathologic condition among ageing men and a leading cause of bladder outlet obstruction (BOO) and lower urinary tract symptoms (LUTS). Approximately 50% of men will develop pathological evidence of BPH over the age of 50 years, and this number increases by 10% per decade and reaches 80% at the eighth decade of age^1^. BPH is a histopathological diagnosis that encompasses microscopic (histological) BPH and macroscopic BPH. Microscopic BPH is characterized by an increased number of epithelial and stromal cells within the periurethral and transition zones, derived mainly from an imbalance between regulative factors of cell death and cell proliferation. Macroscopic BPH is characterized by enlargement of the prostate volume which develops as benign prostatic enlargement (BPE)^2^. The pathogenesis of BPH is multifactorial and largely unknown. Ageing is the predominant factor in development of BPH^3^. Androgen and genetic predisposition play essential and permissive roles^4^,^5^. In addition, recent findings have highlighted the key roles of obesity^6^,^7^, hormonal alterations^8^ and metabolic syndrome^9^ in BPH and LUTS. It has recently been suggested that BPH is a systematic metabolic disease or an obesity-related disease in aging men^10^,^11^. Although considerable progress has been made, the underlying molecular mechanisms of the relationship between BPH and obesity are not yet fully understood.

Adiponectin, a 30 kDa adipokine produced and secreted by adipocytes, is composed of an N-terminal collagen-like sequence and a C-terminal globular region^12^. It exerts multiple protective effects on various cell types, such as insulin-sensitizing action, anti-inflammation, anti-proliferation, anti-atherosclerotic action and suppression of carcinogenesis^13^,^14^. Adiponectin is a relatively abundant serum protein in human, having a physiological concentration between 0.5 and 30 μg/ml, its levels are decreased in various pathological states including obesity, metabolic syndrome and insulin resistance^15^. Previous studies have demonstrated the structures and functions of adiponectin receptors 1 and 2 (AdipoR1 and AdipoR2), which contain a seven-transmembrane domain with an internal N-terminus and an external C-terminus that are topologically distinct from G-protein-coupled receptors^16^,^17^. AdiopR1 and AdipoR2 act as receptors for adiponectin and mainly mediate the activation of 5′ adenosine monophosphate-activated protein kinase (AMPK), peroxisome proliferator-activated receptor-α (PPARα) and p38 mitogen-activated protein kinase (p38-MAPK), regulating glucose and lipid metabolism, cell proliferation and apoptosis^13^,^14^,^18^. In addition, some studies have provided evidence that lower adiponectin levels are associated with an increased risk of BPH and prostate cancer^19^,^20^. These studies led us to hypothesize that adiponectin deficiency could be a potential pathogenic mechanism linking BPH with obesity.

In this study, we assessed the mechanisms underlying the association between obesity and BPH. First, we investigated the biological effects of adiponectin and its receptors on prostatic epithelial and stromal cells. Then, we explored the possible signalling pathway. Finally, we established the emergence of microscopic BPH due to deficiency of adiponectin in an obesity mouse model.

## Results

### Abundant expression of AdipoR1 in human prostatic tissues and cells

**Figure 1:**
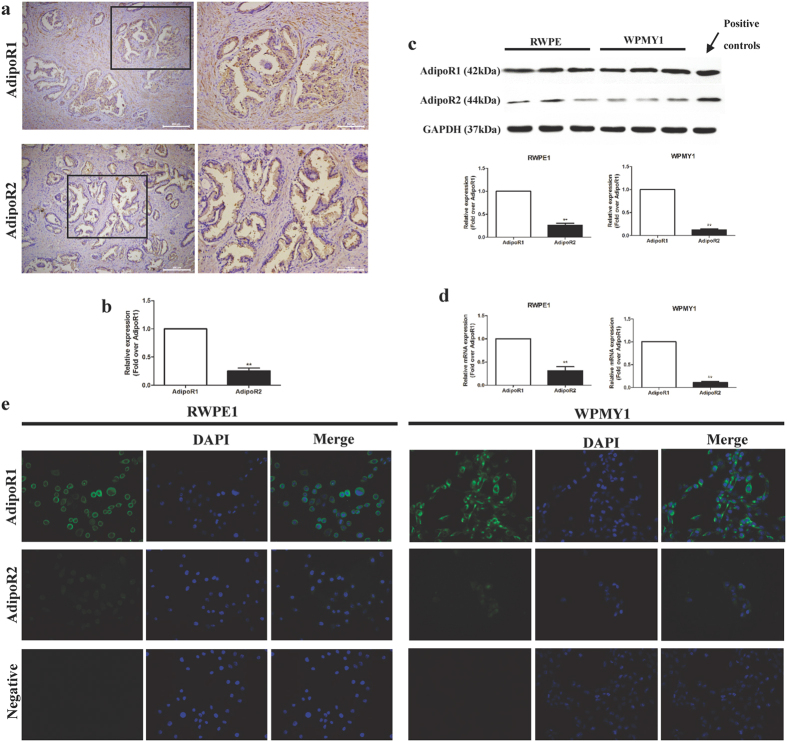
Expression of adiponectin receptors in prostatic tissues and cells. (**a**) IHC staining for AdipoR1 and AdipoR2 on human postoperative BPH tissue samples. Scale bar, 100 μm or 200 μm. (**b**) Semi-quantitation based on the average of optical density (AOD) measured by Image J software (n = 5, Student’s t-test, **p < 0.01). (**c**) WB analysis of adiponectin receptors (AdipoR1 and AdipoR2) expression on RWPE1 and WPMY1 cells. Positive controls were skeletal muscle tissue extract for AdipoR1 and liver extract for AdipoR2. The results were adjusted by GAPDH expression and quantitated as described in the Methods section (n = 3, Student’s t-test, **p < 0.01). Full-length bolts are presented in Supplementary Fig. S3. (**d**) mRNA expression levels of AdipoR1 and AdipoR2 in RWPE1 and WPMY1 cells were determined by RT-PCR. The results were adjusted by β-actin and quantitated by the 2^−ΔΔCt^ method (n = 3, Student’s t-test, **p < 0.01). (**e**) Immunofluorescence staining (green) for AdipoR1 and AdipoR2 in RWPE1 and WPMY1 cells, DAPI staining (blue) for nucleus. Magnification, ×400.

Adiponectin exerts its pleiotropic functions by binding to its receptors (AdipoR1 and AdipoR2), which mediate various downstream signalling pathways^13^,^14^,^16^,^18^. Previous studies have indicated that AdipoR1 and AdipoR2 are expressed on prostate cancer cell lines (PC3, DU145 and LNCAP)^21^,^22^. To investigate the role of adiponectin receptors in normal prostate cells, we detected the mRNA and protein expression of adiponectin receptors in normal prostatic epithelial and stromal cell lines (RWPE1 and WPMY1) by reverse transcription PCR (RT-PCR), immunoblotting and immunofluorescent analysis. RWPE1 and WPMY1 cells had a larger extent of expression of AdipoR1 than of AdipoR2 at both the mRNA and protein levels (Fig. 1c,d), and AdipoR1 was abundantly expressed on the membrane of RWPE1 and WPMY1 cells (Fig. 1e). In agreement with the *in vitro* results, AdipoR1 was expressed approximately five-fold higher than AdipoR2 in human BPH tissues (p < 0.01). Furthermore, AdipoR1 was expressed in both glandular epithelium and prostate stroma (Fig. 1a,b). These findings indicated that adiponectin and AdipoR1 might exert some physiological or pathological effects in the prostate.

### Adiponectin inhibits growth factor mediated proliferation of prostatic epithelial and stromal cells *in vitro*

**Figure 2:**
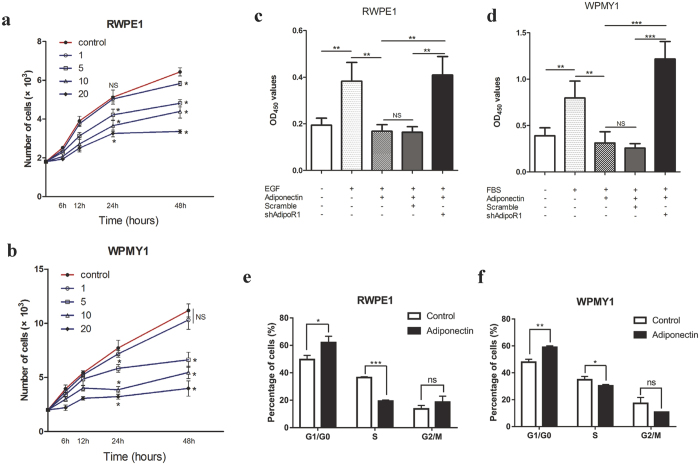
Adiponectin inhibits growth factor-mediated proliferation of prostatic epithelial and stromal cells. (**a**,**b**) CCK-8 proliferation analysis of RWPE1 and WPMY1 cells cultured in regular growth medium, with 0, 1, 5, 10 or 20 μg/ml human recombinant adiponectin treatment. QD_450_ values were converted to cell numbers according to the standard curve. (one-way analysis of variance followed by Dunnett’s post-tests; n = 5; *p < 0.05 versus control, NS, not significant). (**c**,**d**) Cells were cultured with 10 ng/ml EGF or 10% FBS and treated with 10 μg/ml APN or the same volume of PBS. CCK-8 proliferation analysis was performed after 24 h of incubation. The results were expressed as the mean ± s.d. of three independent experiments (one-way analysis of variance followed by Bonferroni post-tests; ***p < 0.001, **p < 0.01, NS, not significant). (**e**,**f**) Cells were treated with 10 μg/ml adiponectin for 12 h or the same volume of PBS as a control after starvation. Then, constituents of the cell cycle were determined by flow cytometry (Student’s t-tests; n = 3; ***p < 0.001, **p < 0.01, *pp < 0.05, NS, not significant).

Numerous studies have demonstrated that adiponectin has anti-proliferation effects on many cells including hepatic stellate cells, vascular smooth muscle cells, endothelial cells and different cancer cell lines^23^,^24^,^25^. CCK-8 analysis showed that, as expected, adiponectin inhibited the growth of RWPE1 and WPMY1 in a dose-dependent manner (Fig. 2a,b). Cells grew faster in the presence of epidermal growth factor (EGF) or foetal bovine serum (FBS) (p < 0.01). In addition, adiponectin treatment abrogated the EFG- or FBS-induced growth-promoting effect (p < 0.01, Fig. 2c,d). However, in the absence of EGF or FBS, the anti-proliferation effect of adiponectin attenuation might be due to the low multiplying rate of cells in the basal culture conditions (see Supplementary Fig. S1). As described in the Methods section, we performed a knockdown of AdipoR1 to mimic the adiponectin deficiency *in vitro* by lentiviral-vector-mediated RNA interference. As shown in Fig. 2c and d, AdipoR1 deficiency resulted in recurrence of the growth-promoting effect of EFG and FBS. These results indicated that adiponectin has an anti-proliferation effect on prostatic epithelial and stromal cells, and cells in the condition of adiponectin deficiency are more susceptible to growth-promoting factors such as EGF and FBS.

### Adiponectin blocks the cell-cycle progression of prostatic epithelial and stromal cells

**Figure 3:**
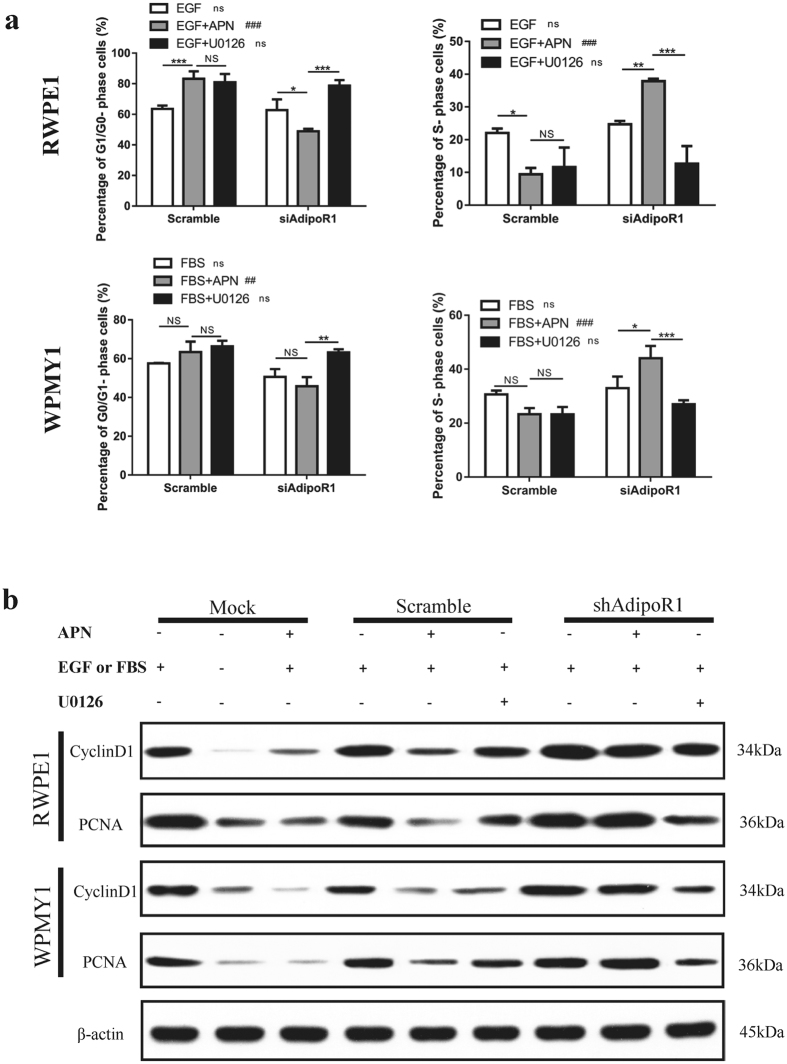
Adiponectin arrests prostatic cells in G_0_/G_1_ phase. (**a**) RWPE1 and WPMY1 cells were treated with the indicated conditions. Then, the cell cycle distribution was analysed with ModFit LT software (V4 1.7, ME). The results are expressed as the mean ± s.d. of three independent experiments (one-way analysis of variance followed by Bonferroni post-tests; ^###^p < 0.001, ^##^p < 0.01, ns, not significant, shAdipoR1 versus scramble; ***p < 0.001, **p < 0.01, *p < 0.05, NS, not significant). (**b**) Cellular extracts were analysed for the expression of cyclinD1 and PCNA after the indicated treatment. The experiment was performed twice with similar results. Full-length bolts are presented in Supplementary Fig. S3. Illustration: shAdipoR1, knockdown of AdipoR1; Scramble, control for shAdipoR1; Mock, untransfected cells; APN, treatment with 10 μg/ml adiponectin for 12 h; U0126, 10 μM pretreated for 2 h; EGF or FBS: supplement of 10 ng/ml EGF for RWPE1 or 10% FBS for WPMY1.

The results of the cell proliferation analysis prompted us to investigate the effect of adiponectin on the cell cycle. In a comparison of adiponectin-treated cells with control cells as indicated in Fig. 2e and f, the average proportion of G0/G1 phase increased and the average proportion of S phase decreased in both RWPE1 and WPMY1 cells. To further investigate the mechanisms of adiponectin arrest of the cell cycle, we performed AdipoR1 knockdown and treated the cells with different conditions as shown in Fig. 3a. U0126 is a highly selective inhibitor of MEK1/2 which competitively binds with mitogen-activated protein kinase/extracellular signal-regulated kinases kinase (MEK), inhibits phosphorylation of extracellular signal-regulated kinases (ERKs) and has been reported to block growth factor-mediated cell survival and cell-cycle progression^26^,^27^. The results showed that adiponectin had an effect similar to that of U0126, blocking cell-cycle progression. More importantly, AdipoR1 deficiency led to the facilitation of the G1/S-phase transition induced by EGF or FBS.

Next, we used immunoblotting to examine protein markers of proliferation and cell cycle (Fig. 3b). The addition of adiponectin markedly suppressed the expression of cyclinD1 and proliferating cell nuclear antigen (PCNA), in contrast to the roles of EGF and FBS. CyclinD1 is a regulatory protein of the cell cycle that dimerizes with cyclin-dependent kinase 4/6 (CDK4/6) to regulate the transition from G1 to S phase. These findings suggest that cyclinD1 might be one of the targets for adiponectin. These results reveal that adiponectin effectively arrests prostate cells in the G_0_/G_1_ phage and inhibits entry into S phase. In the condition of adiponectin deficiency, the adiponectin-mediated cell-cycle blocking effect is abrogated, the EGF- or FBS-mediated cell-cycle advancing effect is strengthened, and the cells show a greater replicative potential.

### Adiponectin stimulates apoptosis of prostatic epithelial and stromal cells by activating caspase 3 *in vitro*

**Figure 4:**
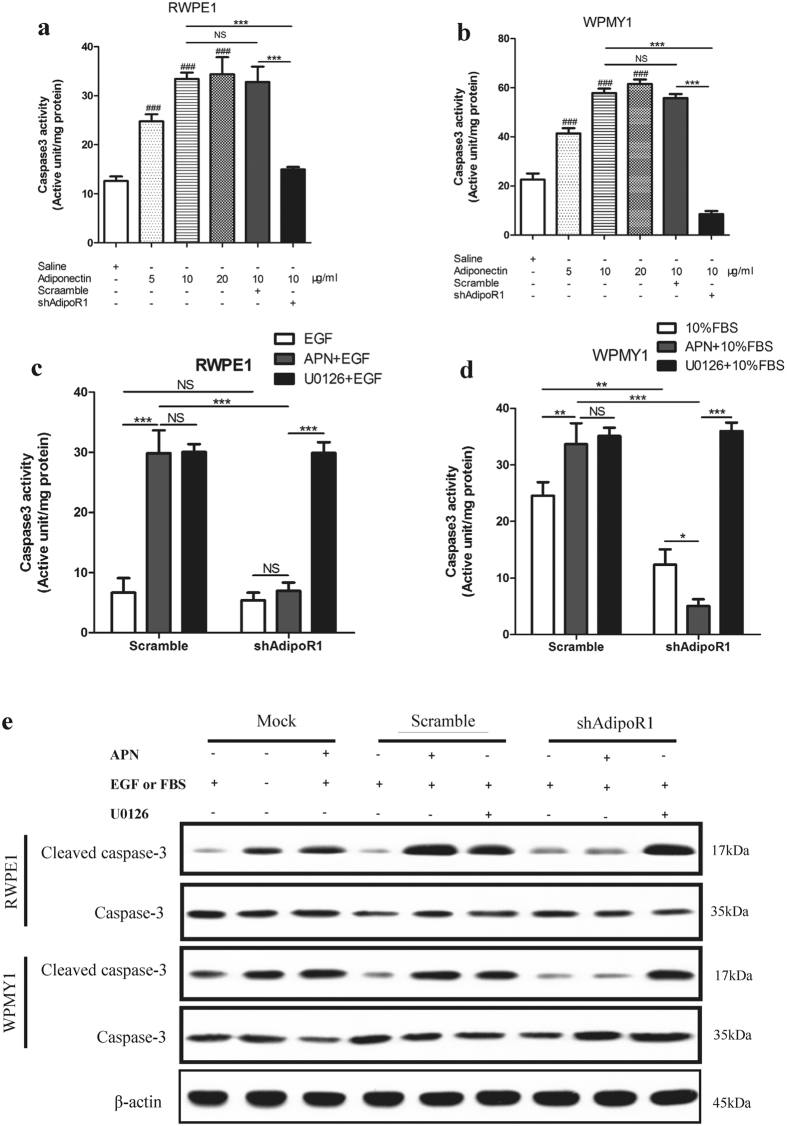
Adiponectin promotes apoptosis of prostatic epithelial and stromal cells by regulating the activation of caspase3. (**a**,**b**) Caspase3 activity assay of RWPE1 and WPMY1 cultured in regular growth medium (control), with 0, 5, 10 or 20 μg/ml human recombinant adiponectin treatment for 12 h, the lentivirus-transfected cells (RWPE1-scramble, RWPE1-shAdipoR1, WPMY1-scramble and WPMY1-shAdipoR1) were treated with 10 μg/ml adiponectin for 12 h. Caspase3 activity is the active unit of caspase3 per unit weight (U/g). The results are presented as the means ± s.d. of three independent experiments (One-way analysis of variance followed by Bonferroni post-tests; ^###^p < 0.001 versus control, ***p < 0.001, NS, not significant). (**c**,**d**) Transfected cells were treated with the indicated conditions before the caspase 3 activity analysis. The results are presented as the means ± s.d. of three independent experiments (one-way analysis of variance followed by Bonferroni post-tests; ***p < 0.001, **p < 0.01, *p < 0.05, NS, not significant). (**e**) Cellular extracts were analysed for the expression of caspase 3 and cleaved-caspase 3 after the indicated treatment by immunoblotting. The experiments were performed twice with similar results. Full-length bolts are presented in Supplementary Fig. S3.

Caspase activation is an important step of apoptotic induction. Adiponectin has been reported to induce a cascade activation of caspase 3^25^,^28^,^29^. To determine the apoptosis induced by adiponectin, we assessed the activation of caspase 3 in RWPE1 and WPMY1 cells. We found that the addition of adiponectin significantly strengthened the activity of caspase 3 in a dose-dependent manner in RWPE1 and WPMY1 cells (Fig. 4a,b). Second, scramble cells and AdipoR1-knockdown cells were cultured in growth medium supplemented with 10% FBS or 10 ng/ml EGF and treated with 10 μg/ml adiponectin or 10 μM U0126 or left untreated. U0126, as noted above, has been reported to promote the activation of caspase 3^26^,^30^. The caspase 3 activity assay (Fig. 4c,d) showed that cells cultured with EGF or FBS had lower levels of caspase 3 activation. Adiponectin treatment promoted activation of caspase 3, and EGF- or FBS-induced anti-apoptotic effect was abrogated by adiponectin. Knockdown of AdipoR1 blocked adiponectin-induced caspase 3 activation but did not influence U0126-induced caspase 3 activation. We obtained the same results with protein expression of cleaved-caspase 3 and caspase 3 by immunoblotting (Fig. 4e).

Next, we examined the levels of Bax and Bcl2 protein expression with adiponectin treatment in different concentrations (see Supplementary Fig. S2a and b). Bax, a pro-apoptotic protein, was obviously upregulated in both RWPE1 and WPMY1 cells after adiponectin treatment. In contrast to Bax, expression of Bcl2 (an anti-apoptotic protein) was downregulated after adiponectin treatment, but its downregulation was less obvious. In general, adiponectin treatment for 6 hours caused a significant increase in the ratio of Bax/Bcl2 protein, indicating that adiponectin promotes apoptosis of the prostate cells through Bax and caspase 3.

### Role of the MEK-ERK-p90RSK axis in mediating adiponectin effects on prostate cells

**Figure 5:**
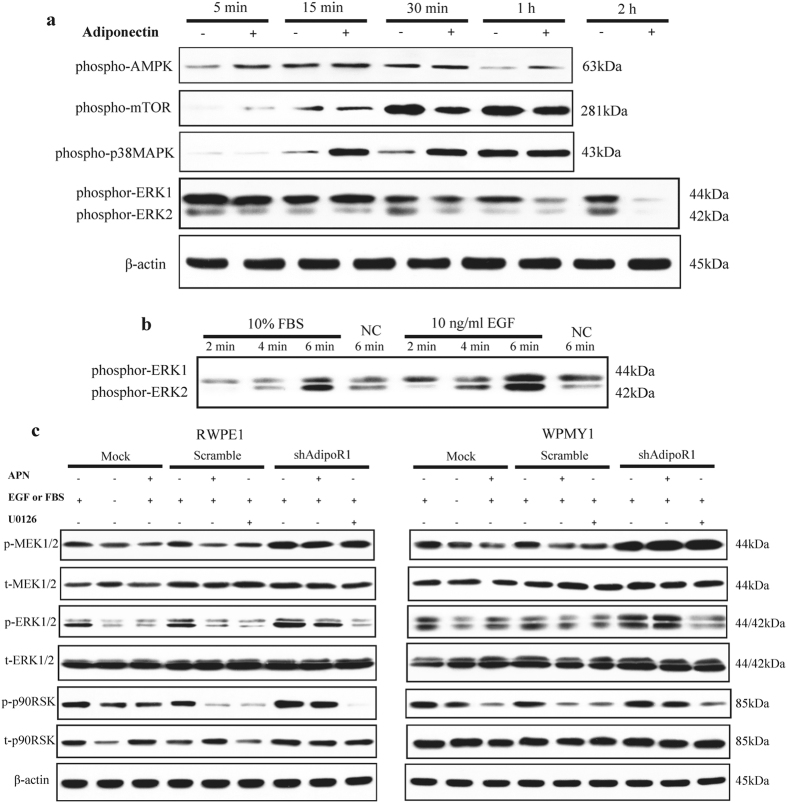
Adiponectin negatively regulates the MEK-ERK-p90RSK axis. (**a**) RWPE1 cells were cultured in growth medium and treated with or without 10 μg/ml human recombinant adiponectin for the indicated times, then the cellular extracts were analysed by immunoblotting for expression of the indicated proteins. (**b**) Cells were starved for 24 h and incubated for 2, 4 or 6 min with supplement of 10 ng/ml EGF for RWPE1 or 10% FBS for WPMY1. NC, negative control treated with PBS. Then, immunoblotting was performed to determine the expression of phospho-ERK1/2. (**c**) RWPE1 and WPMY1 cells were treated with the indicated conditions for 2 h, followed by immunoblotting for the indicated proteins. All immunoblotting experiments were performed twice with similar results. Full-length bolts are presented in Supplementary Fig. S3. Illustration: shAdipoR1, knockdown of AdipoR1; Scramble, control for shAdipoR1; Mock, untransfected cells; APN, treatment with 10 μg/ml adiponectin for 2 h; U0126, treatment with 10 μM U0126 for 2 h; EGF or FBS: supplement of 10 ng/ml EGF for RWPE1 or 10% FBS for WPMY1.

Previous studies have identified several signalling pathways involved in adiponectin signal transduction. APPL1 was the first molecule identified as directly interacting with AdipoR1, which primarily leads to activation of AMPK, PPARα and p38-MAPK by interacting with Rab5^29^,^31^,^32^,^33^. p90 ribosomal S6 kinase (p90RSK) is a downstream effector of ERK, a serine/threonine kinase member of the S6 ribosomal kinase family. It is known to regulate cell proliferation, apoptosis, the cell cycle, mRNA translation, tumour invasion and metastasis and other signalling pathways^34^,^35^. In both RWPE1 and WPMY1 cells, we found that adiponectin rapidly promoted phosphorylation of AMPK (within 5 min) and p38-MAPK (within 15 min) and later inhibited phosphorylation of the mechanistic target of rapamycin (mTOR) (30 min) and ERK (1 hour) (Fig. 5a). In addition, EGF and FBS rapidly activated ERK signalling in prostate cells (Fig. 5b).

We further investigated the downstream event of ERK signalling. Similarly to previous studies^24^,^36^,^37^, we found that phosphorylation of MEK1/2, ERK1/2 and p90RSK induced by EGF or FBS was significantly attenuated by adiponectin treatment. Knockdown of AdipoR1 facilitated phosphorylation of the MEK-ERK-p90RSK axis induced by EGF or FBS (Fig. 5c). These results suggest the involvement of MEK-ERK-p90RSK-axis downregulation in adiponectin-AdipoR1 effects on prostatic epithelial and stromal cells. Furthermore, abrogation of AdipoR1 leads to enhancement of ERK signalling.

### Microscopic BPH induced by a high-fat diet (HFD) in a mouse model was ameliorated by adiponectin supplementation *in vivo*

**Figure 6:**
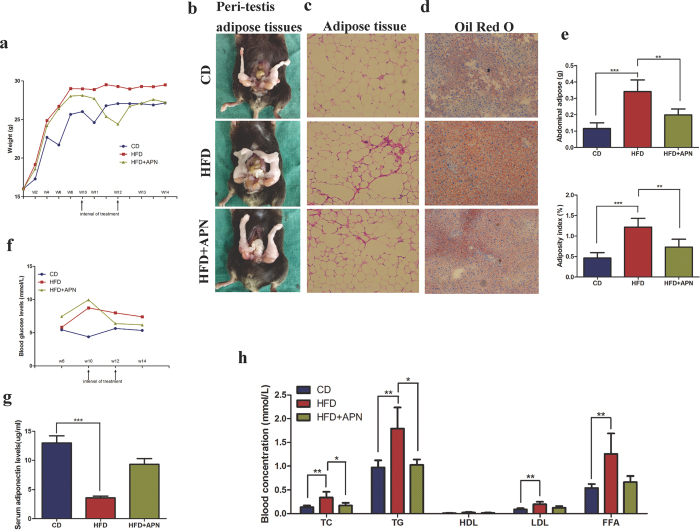
HFD resulted in obesity and metabolic disorders in mice. C57BL/6 mice were fed a low-fat control diet (CD) or a high-fat diet (HFD) for 14 weeks with or without mouse recombinant adiponectin treatment (APN) as described in the Methods section. (**a**) Weight was monitored for the indicated time points. (**b**) Photograph of peri-testis adipose tissues. (**c**) H&E staining for mouse adipose tissues ( ×100 magnification). (**d**) Oil Red O staining for mouse liver tissues to determine the lipid levels in hepatocytes (×200 magnification). (**e**) Abdominal adipose was collected and weighed at the end of 14 weeks. The adiposity index was calculated as the total adipose tissue weight (**g**) divided by the body weight (**g**). (One-way analysis of variance followed by Bonferroni post-tests, n = 5, ***p < 0.001, **p < 0.01). (**f**) Monitoring results of blood glucose. (**g**) Serum adiponectin levels were measured by an ELISA (Student’s t-test, n = 5, ***p < 0.001). (**h**) Blood lipid levels were determined by ADVIA 2400 Biochemistry Analyzer (Siemens). The results are shown as the mean ± s.d. (Kruskal-Wallis nonparametric test, n = 5, **p < 0.01, *p < 0.05). Abbreviations: TC, total cholesterol; TG, triglyceride; HDL, high density lipoprotein; LDL, low density lipoprotein; FFA, free fatty acid.

To investigate the role of adiponectin in the relationship between obesity and BPH, we used a mouse model of HFD induced obesity as described in the Methods section. After 14 weeks, the HFD mice exhibited an obvious increase in body weight and blood glucose compared with the mice on the control diet (CD) (Fig. 6a,f). In addition, the HFD caused an increase in body weight and intra-abdominal adipose weight (Fig. 6b,e). Haematoxylin and eosin (H&E) staining showed that the HFD also resulted in the enlargement of adipocytes and inflammatory infiltration in the adipose stroma (Fig. 6c). We also found that the HFD caused hepatocellular steatosis and lipid metabolic disorders that could be partly alleviated by adiponectin treatment (Fig. 6d,h).

**Figure 7:**
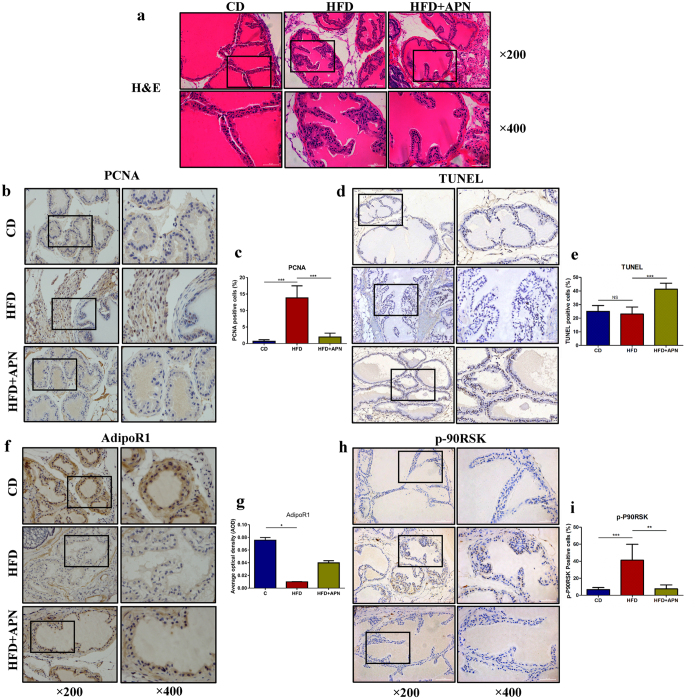
Adiponectin supplement protects prostate from microscopic BPH induced by HFD. C57BL/6 mice were fed a low-fat control diet (CD) or a high-fat diet (HFD) for 14 weeks with or without mouse recombinant adiponectin treatment (APN) as described in the Methods section. (**a**) Histopathological analysis with H&E staining showing characteristics of prostatic glands and stroma (×200 and ×400 magnification). (**b**,**d**,**f**,**h**) TUNEL assay and immunohistochemical staining with antibodies against PCNA, p-P90RSK and AdipoR1 ( ×200 and ×400 magnification). (**c**,**e**,**g**,**i**) The results were presented as the average percentage of positive cells over the total counted cells. Expression of AdipoR1 was presented as the average of optical density (AOD) (Kruskal-Wallis nonparametric test, n = 5, ***p < 0.001, **p < 0.01, *p < 0.05).

Previous studies based on rat and rabbit models revealed that an HFD led to prostatic alterations, including prostate enlargement, inflammatory infiltration and stromal fibrosis^38^,^39^. In our studies, we did not find macroscopic BPH (increase in prostate size and weight), partly because of the unlocalized and nonenveloped distribution of mouse prostate tissues, it was difficult to completely separate the prostate from peripheral tissues. However, we found signs of obvious microscopic BPH, such as increases in the number of glandular epithelial and stromal cells and formation of secondary papillary branches (Fig. 7a). Consistent with previous studies^40^,^41^, we found that an HFD resulted in adiponectin deficiency, including downregulation of AdipoR1 and reduction in serum adiponectin levels (Figs 6g and 7f,g). As expected, the supplementation of adiponectin showed protective effects against an HFD, alleviating mouse obesity and microscopic BPH.

As shown in Fig. 7b,c,d and e, the prostates of the HFD mice showed similar apoptosis status but more PCNA^+^ proliferative cells compared with the prostates of the CD mice, and the prostates of the adiponectin treatment group (APN) had fewer PCNA^+^ proliferative cells but more apoptotic cells compared with the HFD group. In addition, we performed IHC staining with antibodies against phospho-p90RSK and found that, in agreement with the *in vitro* results, the HFD mice showed higher expression of p-p90RSK in the prostate compared with the CD mice, and adiponectin treatment significantly decreased the expression of p-p90RSK (Fig. 7h,i). These results provide evidence that adiponectin deficiency probably contributes to obesity-related microscopic BPH.

## Discussion

Recent epidemiological studies have closely linked BPH with obesity^6^,^7^. Adiponectin has received much attention because of its inverse association with the consequences of obesity^13^. Considering that few studies have determined the expression of adiponectin receptors in the human prostate^21^, we performed a series of experiments and found greater expression of AdipoR1 than AdipoR2 in prostatic glandular epithelium and stroma. Furthermore, AdipoR1, but not AdipoR2, was plentifully located in the membrane of prostatic epithelial and stromal cells (Fig. 1). *In vitro*, we found multifunctional effects of adiponectin on human normal prostatic epithelial and stromal cells inhibiting proliferation, promoting apoptosis and blocking progression of G1/S phase. We also found attenuated adiponectin effects in the absence of growth factors (GFs) and aggravated adiponectin effects in the presence of GFs. More importantly, knockdown of AdipoR1 caused failure of adiponectin effects and indirectly amplified the GFs effects. We believe that adiponectin effects protect prostate cells from excessive proliferation and maintains the balance of cell numbers. Thus, adiponectin, together with GFs, can be regarded as the “stability-holder” of cell numbers. Cells tend to have stronger replicative capacity in the condition of adiponectin deficiency, this capacity is limited and controllable. Although several studies have found correlative evidence between adiponectin deficiency and cancers^25^, including breast cancer^42^, hepatocellular carcinoma^43^ and prostate cancer^44^. Additional studies are needed to determine whether adiponectin deficiency contributes to the limitless replicative potential of cancer cells.

Cell growth and death are determined primarily by the extracellular environment. In the prostatic microenvironment, several GFs play an essential role in cell growth and survival in a paracrine or endocrine manner^45^, and GFs are activators of ERK signalling^26^,^42^,^46^,^47^. The absence of GFs or serum deprivation in culture medium of prostate cells results in apoptosis and weakness of proliferation. In our studies we found that adiponectin attenuated ERK signalling (the MEK-ERK-p90RSK axis), which is activated by EGF or FBS. This finding suggests that ERK signalling acts as a suppressive downstream target of adiponectin. Indeed, the knockdown of AdipoR1 abrogated the suppressive effect of adiponectin on ERK signalling. Moreover, in contrast to the expression of AdipoR1, p90RSK (a downstream effector of ERK) was upregulated in human BPH tissues and obese mice. Our results suggest a link between adiponectin-AdipoR1 signalling and ERK signalling. However, evidence has been found that activation of AMPK negatively modulates the ERK signalling^37^,^48^. The available data is not yet sufficient to confirm whether adiponectin negatively regulates the ERK pathway through activation of AMPK or through an independent pathway. Further studies are needed to elucidate the specific interaction between adiponectin-AdipoR1 and the ERK signalling pathway.

To our knowledge, this study is the first to consider both adiponectin deficiency (decrease of serum adiponectin levels and downregulation of AdipoR1) and BPH in an HFD mouse model. We found that the prostates of HFD-mice were characterized by microscopic hyperplasia, decreased expression of AdipoR1 and increased expression of p-P90RSK. Importantly, HFD-induced prostatic alternations were counteracted by extra adiponectin supplements, suggesting that correcting adiponectin deficiency prevents the development of HFD-induced BPH. However, we do not yet know whether the predominant mechanism is direct or indirect yet.

**Figure 8:**
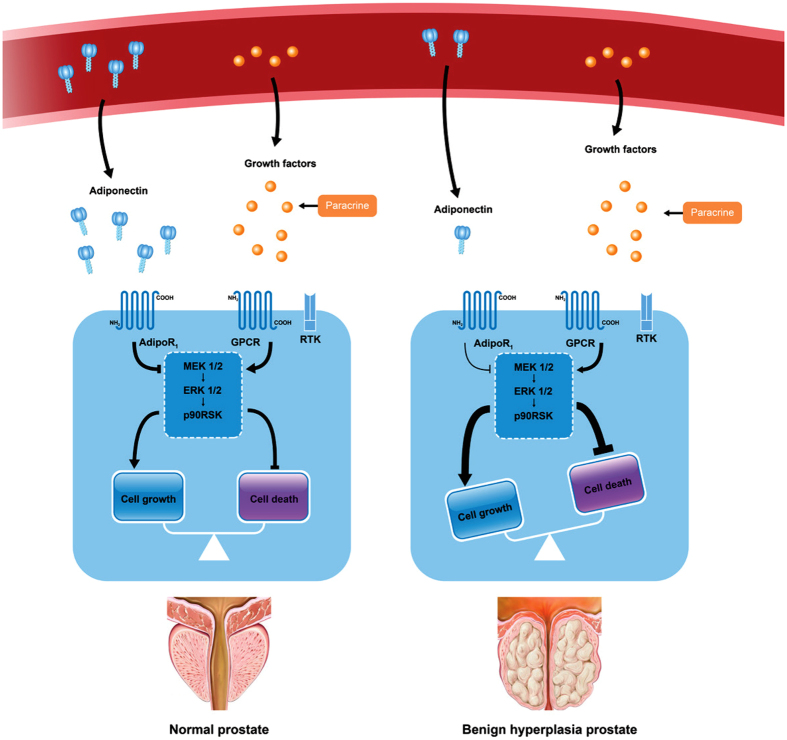
A model for adiponectin deficiency-induced facilitation of the MEK-ERK-p90RSK axis and aggravation of cell growth imbalance. Abbreviations: AdipoR1, adiponectin receptor 1; GPCR, G-protein-coupled receptors; RTK, receptor tyrosine kinase.

Our findings provide novel insight into the causal role of adiponectin deficiency in the development of microscopic BPH. However, potential limitations should be taken into account when interpreting our study. A major limitation is that the HFD-mouse model is characterized by dyslipidaemia, insulin resistance and increased insulin and leptin levels^49^,^50^. These confounders appear simultaneously and affect one another. Because it is difficult to isolate the effect of adiponectin *in vivo*, the observed results reflect the incorporated effects of adiponectin and other metabolic alternations. Our hypothesis is still strongly supported by the negative association between adiponectin levels and the degree of hyperplasia. A more thorough assessment of metabolic alternations is needed to eliminate confounders. Overall, adiponectin plays a negative regulatory role in maintaining a balance of cell numbers through inhibiting the MEK-ERK-p90RSK axis; adiponectin deficiency occurs in the condition of obesity and leads to abrogation of this negative regulatory effect and imbalance between cell growth and death, further resulting in the development of BPH (Fig. 8).

If our hypothesis of a causal role of adiponectin deficiency in the development of BPH is true, correcting adiponectin deficiency might bring about slowing of the prostate growing rate. In fact, adiponectin levels might be enhanced by increased physical activity, weight loss or dietary changes^51^,^52^. Recent epidemiological studies have reported that life-style factors such as increasing physical activity^53^, bariatric surgery^54^ and intake of more vegetables and less high-fat food^55^ appear to protect against the development of BPH-LUTS. For this reason, our report also implies a hypothesis that lifestyle modifications such as regular physical activity, weight loss and dietary changes protect against the development of BPH perhaps by enhancing the adiponectin effect.

In summary, we have established that adiponectin deficiency results in activation of the MEK-ERK-p90RSK axis which contributes to the association between obesity and BPH. Adiponectin deficiency is a pathological condition that is due to systemic disorders. Hence, our findings suggest that urologists should direct the therapeutic strategies towards the systemic health conditions comprehensively. The findings might also provide evidence to promote a low-fat diet, weight loss, physical activity and other lifestyle modifications as early prevention strategies for high-risk populations to reduce the growth rate of the prostate, delay or prevent the occurrence of BPH/LUTS and metabolic disorders, and ultimately, achieve healthy aging.

## Methods

### Antibodies and regents

Antibodies of Cleaved-caspase 3 (9661), caspase 3 (9662), PCNA (2586), phospho-MEK1/2 (9154), phospho-p90RSK (11989), phospho-MSK1 (9595), phospho-p38 MAPK (9211), Bax (2774) and Bcl-2 (2872) were obtained from Cell Signaling Technology (CST, MA, USA). Antibodies of AdipoR1 (ab126611), AdipoR2 (ab189446), p90RSK (ab32114), cyclinD1 (ab134175), total-ERK1/2 (ab184699), phospho-ERK1/ (ab76299), total-MEK1/2 (ab178876), phospho-AMPK (ab133448) and phospho-mTOR (ab109268) were obtained from Abcam (Cambridge, UK).

Recombinant human adiponectin (1065-AP), recombinant mouse adiponectin (5095-AC) and recombinant human epidermal growth factor (236-EG) were obtained from R&D System. MEK1/2 inhibitor U0126 was obtained from CST (9911).

### Human samples

To investigate the expression of adiponectin receptors in human prostate tissues, we collected postoperative prostate specimens from 5 informed BPH patients who were undergoing operative treatment in our hospital. Diagnosis of BPH was based on a total prostate volume (TPV) ≥30 ml and an International Prostate Symptom Score (IPSS) ≥7. The specimens were formalin fixed and paraffin embedded. Then, immunohistochemical staining was performed with antibodies to AdipoR1 (1:200), AdipoR2 (1:200). Because the staining of adiponectin receptors was distributed mostly in the membrane and cytoplasm, it was difficult to distinguish positive-stained cells from tissues. For this reason, the staining extent and intensity were semi-quantitated as the average of optical density (AOD), which was measured using Image-Pro Plus (IPP, version 6.0, Media Cybernetics)^56^. All patients gave written informed consent prior to operation. The study was approved by the Ethics Committee of Shanghai Jiao Tong University School of Medicine and all methods were performed according to the guidelines approved by the ethics committee.

### Cells culture and treatment

The human prostatic epithelial cell line (RWPE1) and human prostatic stromal cell line (WPMY1) were purchased from American Type Culture Collection (ATCC, USA). RWPE1 was grown in prostatic epithelial cell medium (PEpiCM, ScienCell) with a 1% prostatic epithelial cell growth supplement containing various growth factors (PEpiCGS, ScienCell) and a 1% penicillin/streptomycin solution (P/S, ScienCell). WPMY1 cells were grown in high-glucose DMEM (HyClone) supplemented with 10% FBS (HyClone) and 1% penicillin/streptomycin. The cells were cultured at 37 °C with 5% CO_2_ in 100 mm culture dishes. Images were photomicrographed using a DXM1200 digital camera (Nikon).

For some experiments, the cells were pretreated with 10 μM U0126 for 2 h and treated with different concentrations of adiponectin for the indicated time. As previously reported, EGF receptors were expressed on RWPE1 cells, and EGF stimulated the growth of RWPE1 cells through activating the ERK signalling pathway^46^,^47^. Therefore, in our experiments, RWPE1 cells were cultured in PEpiCM without PEpiCGS as a negative control, or treated with 10 ng/ml EGF as positive treatment activating the ERK signalling pathway. However, we found that EGF alone was less effective in stimulating growth of WPMY1 cells according to the cell proliferation assay. The growth of WPMY1 relies primarily on FBS and FBS obviously stimulates phosphorylation of MEK (Fig. 6b). In our studies, WPMY1 cells were cultured in serum-free medium (SFM) as a negative control or supplemented with 10% FBS as positive treatment activating the ERK signalling pathway. In addition, the cells were starved for 12 h before some of the experiments.

### RNA interference and generation of AdipoR1-knockdown cells

The lentiviral vectors containing the shRNA of human AdipoR1 (Lenti-shAdipoR1, sequence 5′-TGGCTCTTTCACACCGTCT-3′) or a scrambled sequence (Lenti-Control) were constructed by Asia-Vector Biotechnology (Shanghai, China). The lentivirus was harvested every 48 and 72 h after being packaged into HEK293T cells. RWPE1 and WPMY1 cells were plated in 6-well plates at a density of 5 × 10^5^ cells per well and cultured for 24 h. The next day, the cells were infected with Lenti-shAdipoR1 or Lenti-Control with 8 μg/ml polybrene (Santa Cruz, CA) for 24 h at a multiplicity of infection (MOI) of 10:1. Next, infected cells were selected with 5 μg/ml puromycin (InvivoGen, USA) for 6 days to obtain stable knockdown cell lines (RWPE-shAdipoR1, RWPE-Scramble, WPMY-shAdipoR1 and WPMY-Scramble). The transfection efficiency was evaluated under a fluorescence microscope. The knockdown efficiency was confirmed by western blotting.

### Cell proliferation assay

Cells were plated in 96-well plates at 2000 cells per well in 100 μl of medium with or without FBS and treated with different concentrations of adiponectin, 10 ng/ml EGF or PBS. At different time points (0 h, 6 h, 12 h, 24 h and 48 h), cell proliferation was analysed by a Cell Counting Kit-8 (CCK-8) assay according to the manufacturer’s instructions (C0037, Beyotime Biotechnology, China). Briefly, 10 μl of CCK-8 solution was added to each well and the cells were cultured at 37 °C for 1 h. The absorbance at 450 nm was measured with a Varioskan Flash Spectral Scanning Multimode Reader (Thermo).

### Caspase 3 activity assay

Cells were plated in 60 mm culture dishes at a density of 1 × 10^6^ cells per dish. Caspase 3 activity was analysed using a caspase 3 activity kit (C1115, Beyotime Biotechnology, China), which is based on the ability of caspase 3 to catalyse the substrate (Ac-DEVD-ρNA) to yellow ρ-nitroaniline (ρNA). Briefly, the cells were lysed (100 μl of lysis buffer per 2 × 10^6^ cells) after being treated with different conditions for 12 h. Next, 50 μl of cell lysate, 40 μl of reaction buffer and 10 μl of caspase 3 substrate were mixed in 96-well microplates and incubated at 37 °C for 2 h. The absorbance at 405 nm was measured with a Varioskan Flash Spectral Scanning Multimode Reader (Thermo). The protein content was determined using the Bradford method (P0006, Beyotime Biotechnology, China). Caspase 3 activity was expressed as the active unit of caspase 3 per unit of weight (U/g).

### Cell cycle analysis

Cells were plated in 60 mm culture dishes at a density of 1 × 10^6^ cells per dish. After being starved for 12 h and treated with the indicated conditions for 12 h, the treated cells were collected and washed twice with cold PBS. Then, the single cell suspension was fixed with 70% cold ethanol for 12 h. Finally, according to the manufacturer’s instructions (C1052, Beyotime Biotechnology, China), the cells were stained with a propidium iodide (PI) mixture for 30 minutes at 37 °C before flow cytometry analysis (Beckman Coulter Flow Cytometer, Krefeld, Germany). The cell cycle distribution was further analyzed with ModFit LT (V4 1.7, Verity Software House, Topsham, ME).

### RNA isolation and RT-PCR

Total RNA was extracted from RWPE1 and WPMY1 cells with TRIzol reagent (Invitrogen, CA) and reverse transcribed using the MMLV system (promega) to obtain cDNA. Quantitative real-time PCR was performed using a Vii 7 Real-Time PCR System (Applied Biosystems, Foster City, CA). The primer sequences for AdipoR1 and AdipoR2 were as previously described^16^. The relative mRNA expression was normalized by β-actin expression and determined using the 2^−ΔΔCt^ method.

### Protein isolation and immunoblotting

Cells were collected and lysed on ice with RIPA Lysis and Extraction buffer (Thermo Scientific) and with Halt Protease and Phosphatase Inhibitor Cocktail (Thermo Scientific). After being centrifuged for 10 min at 10000 *g* at 4 °C, the supernatants were collected and the total protein concentration was determined with a BCA protein assay kit according to the manufacturer’s instructions (P0010S, Beyotime Biotechnology, China). The lysates were loaded and separated by SDS–PAGE gel electrophoresis, and then transferred to PVDF membranes (Millipore). The membranes were blocked with TBS/T buffer containing 5% milk and incubated with primary antibodies against AdipoR1 (1:1000, Abcam), AdipoR2 (1:1000), PCNA (1:2000), cyclinD1 (1:1000), caspase-3 (1:2000), cleaved caspase-3(1:2000), phospho-MEK1/2 (1:1000), total-MEK1/2 (1:1000), phospho-ERK1/2 (1:1000), total-ERK1/2 (1:1000), phospho-p90RSK1/2 (1:1000), total-p90RSK1/2 (1:1000). phospho-p38 MAPK (1:1000), phospho-AMPK (1:1000), phospho-mTOR (1:2000), BAX (1:1000), BCL-2 (1:1000), GAPDH (1:2000) and β-actin (1:2000) overnight at 4 °C. Next, the membranes were washed and incubated with a horseradish peroxidase-conjugated secondary antibody (1:2000, Jackson) at room temperature for 2 h and finally detected by the chemiluminescent method with ECL Western Blotting Substrate (Thermo Scientific). The blots were visualized and analysed with the GelPro Imaging System (CBIO, Beijing, China) and GelPro 1D software version 4.5.

### Immunofluorescence

RWPE1 and WPMY1 cells were plated on Fisherbrand Coverglass (6-well, Thermo Fisher, USA) at a density of 5 × 10^5^ per well. After regular incubation for 24 h, 4% paraformaldehyde was used to fix the cells for 20 min. Then, the cells were blocked with goat serum and incubated with primary antibodies against AdipoR1 (1:200) and AdipoR2 (1:200) overnight at 4 °C. Finally, the slides were incubated with a FITC conjugated secondary antibody (1:100, Boster, Wuhan, China) for 1 h at 37 °C. The nuclei were counterstained with DAPI (C1002, Beyotime Biotechnology, China). The slides were photographed under a BX53 fluorescent microscope (Olympus).

### Animals

A mouse model of HFD induced obesity was used to investigate the underlying relevance between obesity, BPH and adiponectin^57^,^58^. Fifteen male C57BL/6 mice (3 weeks old, 16–17 g), provided by the Animal Center of Ninth People’s Hospital were randomly assigned to three groups (5 mice per group): the control diet (CD) group, which was fed a low-fat control diet (D12450H, Research Diets, New Brunswick, NJ) and treated with normal saline (NS, 0.9% NaCl) intraperitoneally; the HFD group, which was fed an HFD (D12451, Research Diets, New Brunswick, NJ) and treated with NS intraperitoneally; and the adiponectin treatment group (APN) group, which was fed an HFD and treated with mouse recombinant adiponectin intraperitoneally (i.p.) for a total of 14 days (50 μg per mouse once daily) from the 10^th^ week to the 12^th^ week^59^. The diets were matched for protein content, fat content (mainly lard and soybean oil) and sucrose calories (17%), and had the following energy content (% of total calories): the low-fat diet was 10% fat, 20% protein and 70% carbohydrate; the HFD was 45% fat, 20% protein and 35% carbohydrate. The mice were sacrificed at the end of the 14^th^ week, and their prostate, liver and intra-abdominal adipose tissues (peri-testis and peri-renal adipose tissues) were collected and weighted. The adiposity index was calculated as the total adipose tissue weight (g) divided by the body weight (g)^38^. The prostate samples were formalin fixed and paraffin embedded. Blood samples were collected from the orbital plexus and centrifuged at 3000 rpm for 5 min to obtain serum samples and stored at −80 °C before further studies. Blood glucose levels were monitored in the tail vein blood using OneTouch UltraVue (sensitivity was 1.1 mmol/L, Johnson & Johnson, USA)^60^. Blood lipid levels were determined by an ADVIA 2400 Biochemistry Analyzer (Siemens). Serum adiponectin levels were measured using an enzyme-linked immunosorbent assay kit (sensitivity 49 pg/ml, RayBiotech, Norcross, GA, USA). Each sample was tested in triplicate. The inter-assay coefficient of variation was less than 10%, and the intra-assay coefficient of variation was less than 12%.

All mice were housed in a specific pathogen-free facility (temperature, 20–22 °C; humidity, 50–60%) and had free access to food and water in a 12 h light-dark cycle. All animal procedures were approved by the Animal Ethics Committee of Shanghai Jiao Tong University School of Medicine. The methods applied in this study were performed in accordance with the Guide for the Care and Use of Laboratory Animals (NIH pub #90–23).

### Histology and immunohistochemistry

Formalin-fixed, paraffin-embedded prostatic tissues were sectioned at 5 μm. The sections were stained with H&E first to observe the characteristics of histomorphology. Immunohistochemical staining was performed according to the diaminobenzidine staining procedure of the Dako EnVision detection system (Dako, Denmark). Antigen retrieval was processed via the heating method with 10 mM sodium citrate buffer (PH 6.0) for 30 min. Then, primary antibodies of AdipoR1 (1:400), AdipoR2 (1:200), PCNA (1:4000) and phsopho-p90RSK (1:400) were used and diluted with TBS (0.05 M). Negative controls were acquired omitting primary antibodies. The slides were photographed with a DM2500 (LEICA). Membranous staining (for example, AdipoR1 and AdipoR2 IHC staining) was semi-quantitated as the average of optical density (AOD), which was measured using Image-Pro Plus (IPP, version 6.0, Media Cybernetics). The results of nucleus staining (for example, PCNA and p90RSK staining) were presented as the average percentage of positive cells over the total counted cells^61^, and five visual fields under a ×400 magnification were randomly selected to count. The results were evaluated independently by two assessors (C.L. and M.G.) who were blinded to the design and process of the experiments. Mouse liver tissue slides were stained with a lipid (Oil Red O) staining kit according to the manufacturer’s instructions (MAK194, Sigma-Aldrich).

### TUNEL assay

TUNEL assay was performed to detect and quantify apoptosis of paraffin embedded prostate tissues following the manufacturer’s instruction (*In Situ* Cell Death Detection Kit, Roche, USA). Five visual fields under a × 400 magnification were randomly selected to count the positive staining cells by two assessors in a blinded manner independently. The results were presented as the average percentage of positive cells over total counted cells.

### Statistical analysis

The data were expressed as the mean values with standard deviation or numbers with proportions. Comparisons were conducted using Student’s t-test or a nonparametric test (Mann-Whitney U test or Kruskal-Wallis test). Analysis of variance (ANOVA) followed by Bonferroni’s post-test was performed for multiple comparisons. A two-sided p value less than 0.05 was considered statistically significant. All analyses were performed using SPSS, version 21.0 (SPSS Inc, Chicago, IL, USA) and GraphPad Prism 5.0 software (San Diego, CA).

## Additional Information

**How to cite this article**: Fu, S. *et al*. Adiponectin deficiency contributes to the development and progression of benign prostatic hyperplasia in obesity. *Sci. Rep.* **7**, 43771; doi: 10.1038/srep43771 (2017).

**Publisher's note:** Springer Nature remains neutral with regard to jurisdictional claims in published maps and institutional affiliations.

## Supplementary Material

Supplementary Materials
