# Supplementary material for: Adiponectin deficiency contributes to the development and progression of benign prostatic hyperplasia in obesity
Source: Sci Rep. 2017 Mar 3;7:43771. doi: 10.1038/srep43771 (PMC5335662; doi:10.1038/srep43771)
Supplement: Supplementary Materials [file srep43771-s1.pdf]

Supplementary materials

Title: Adiponectin deficiency contributes to the development and progression of benign prostatic hyperplasia in obesity

Authors: Shi Fu, Huan Xu, Meng Gu, Chong Liu, Qiong Wang, Xiang Wan, Yanbo Chen, Qi Chen, Yubing Peng, Zhikang Cai, Juan Zhou & Zhong Wang

Correspondence to: Zhong Wang, Department of Urology and Andrology, Shanghai Ninth People's Hospital, Zhizaoju Road, No. 639, Shanghai, 200011, China. E-mail: zhongwang2000@sina.com; Tel: (86-21)23271007; Fax: (86-21)63136856.

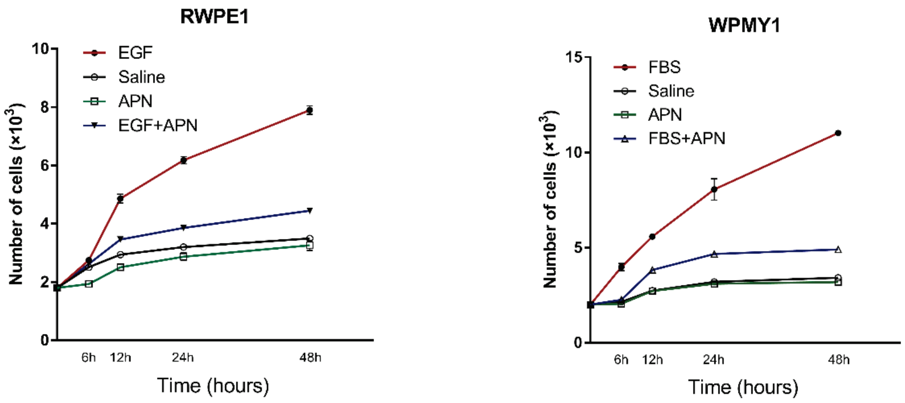

Supplementary figure S1. The cell growth curve of RWPE1 and WPMY1 cells with treatment of the indicated conditions.

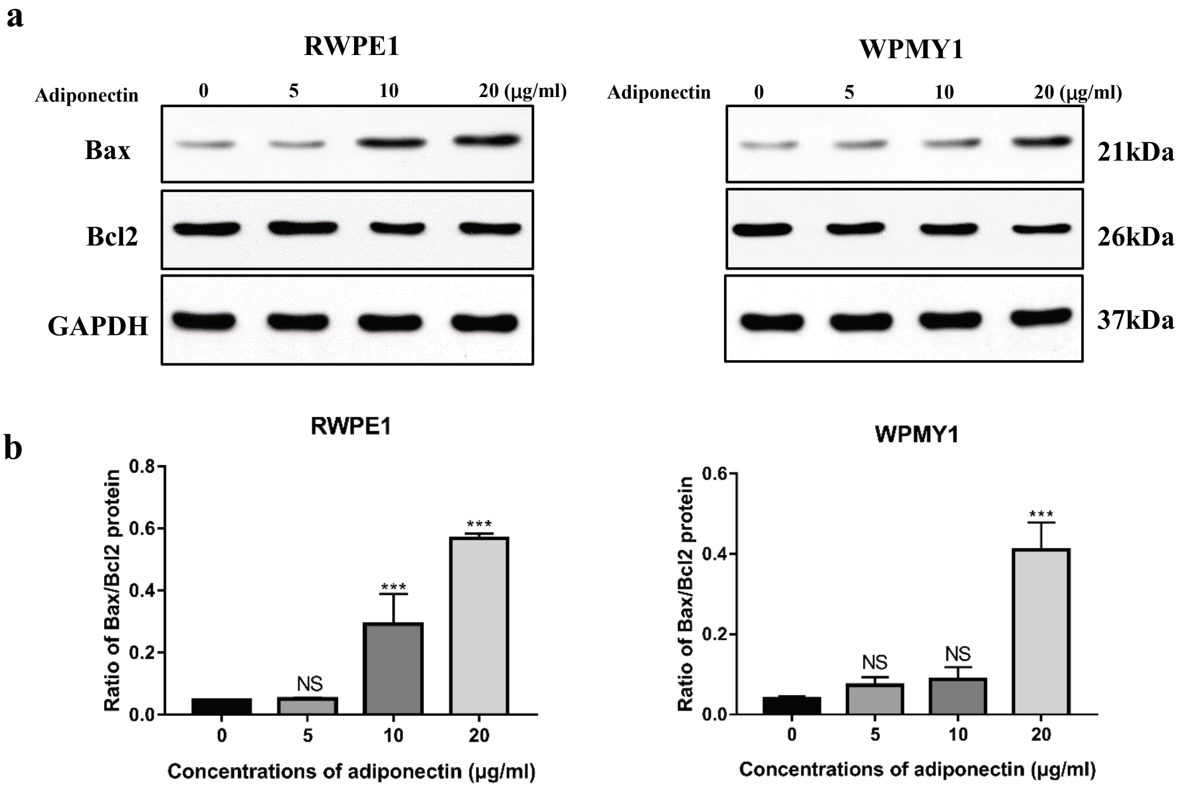

Supplementary figure S2. (**a**) Cells were treated with different concentrations of adiponectin (0, 5, 10 and 20 µg/ml) for 6 h. Then cellular extracts were analysed for the expression of Bax and Bcl2 by immunoblotting. (**b**) The ratio of Bax and Bcl2 was determined. Data are presented as the means ± s.d. of three independent experiments (one-way analysis of variance followed by Dunnett's post-tests; n=3; ***$p<0.001$ versus control, NS, not significant).

Fig. 1

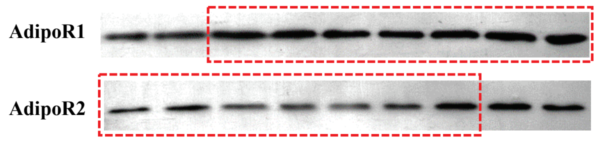

Fig. 3

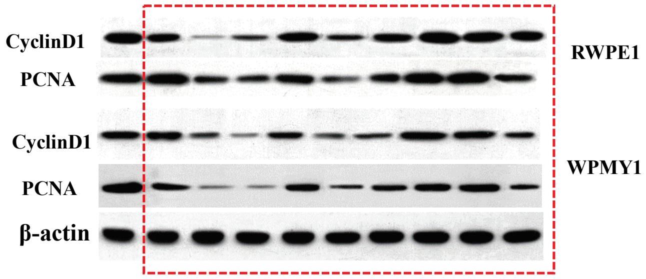

Fig. 5

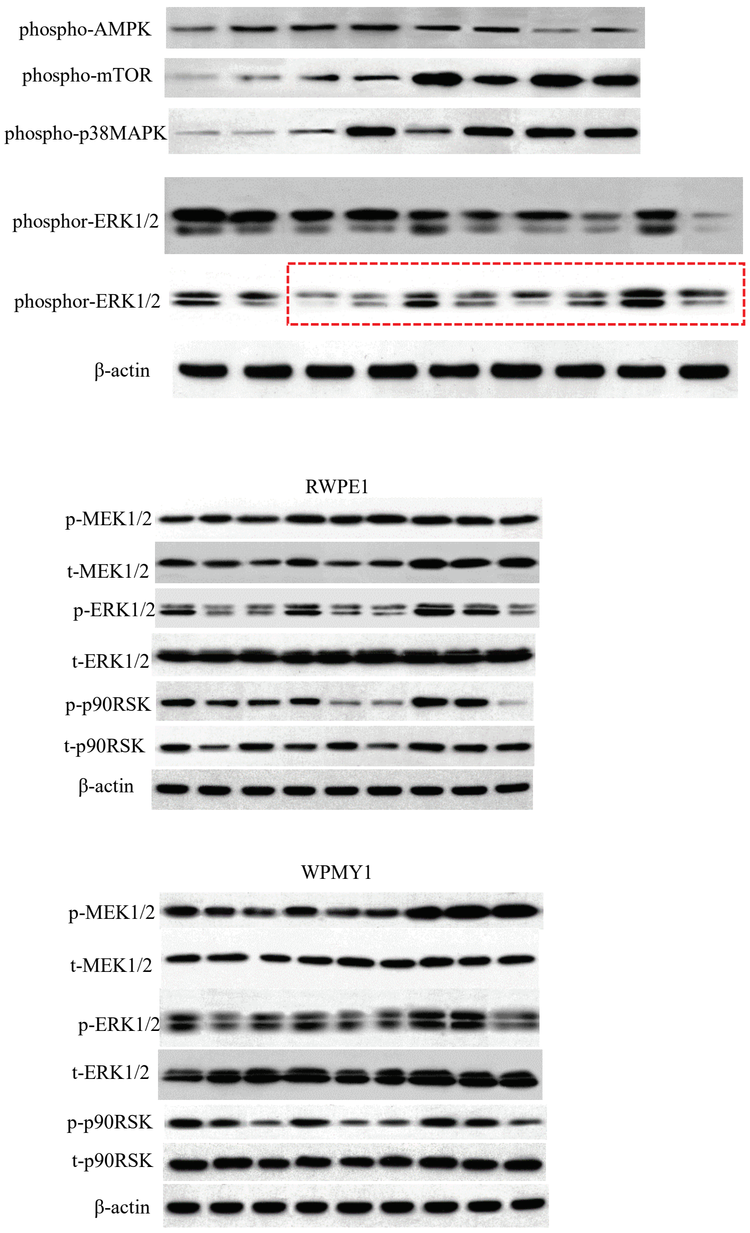

Fig. 4

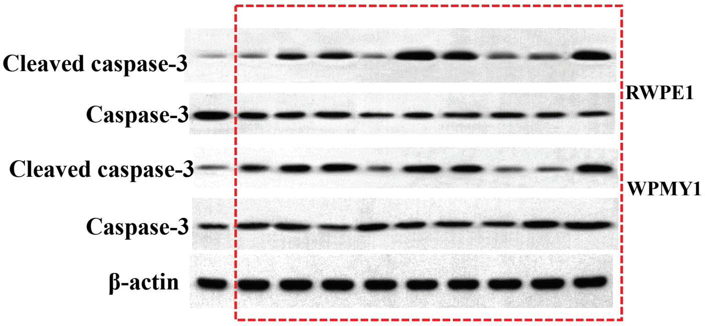

Fig. S2

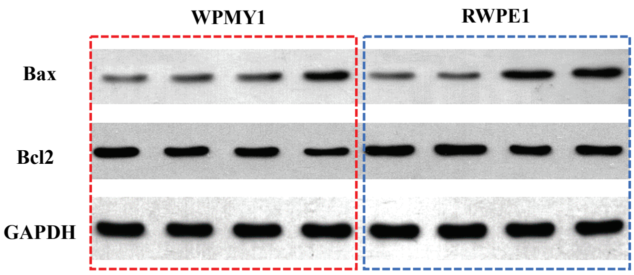

Supplementary figure S3. Full-length blots for some cropped and high-contrast protein bands in the paper.
